# Cell Wall Invertase and Sugar Transporters Are Differentially Activated in Tomato Styles and Ovaries During Pollination and Fertilization

**DOI:** 10.3389/fpls.2019.00506

**Published:** 2019-04-18

**Authors:** Si Shen, Si Ma, Yonghua Liu, Shengjin Liao, Jun Li, Limin Wu, Dewi Kartika, Hans-Peter Mock, Yong-Ling Ruan

**Affiliations:** ^1^School of Environmental and Life Sciences, The University of Newcastle, Callaghan, NSW, Australia; ^2^College of Agronomy and Biotechnology, China Agricultural University, Beijing, China; ^3^Beijing Key Laboratory of Growth and Developmental Regulation for Protected Vegetable Crops, College of Horticulture, China Agricultural University, Beijing, China; ^4^Institute of Tropical Agriculture and Forestry, Hainan University, Haikou, China; ^5^CSIRO Agriculture and Food, Canberra, ACT, Australia; ^6^Leibniz Institute of Plant Genetics and Crop Plant Research, Gatersleben, Germany

**Keywords:** cell wall invertase, fertilization, fruit set, pollination, pollen tube elongation, sugar transporter, sugar utilization, tomato

## Abstract

Flowering plants depend on pollination and fertilization to activate the transition from ovule to seed and ovary to fruit, namely seed and fruit set, which are key for completing the plant life cycle and realizing crop yield potential. These processes are highly energy consuming and rely on the efficient use of sucrose as the major nutrient and energy source. However, it remains elusive as how sucrose imported into and utilizated within the female reproductive organ is regulated in response to pollination and fertilization. Here, we explored this issue in tomato by focusing on genes encoding cell wall invertase (CWIN) and sugar transporters, which are major players in sucrose phloem unloading, and sink development. The transcript level of a major CWIN gene, *LIN5*, and CWIN activity were significantly increased in style at 4 h after pollination (HAP) in comparison with that in the non-pollination control, and this was sustained at 2 days after pollination (DAP). In the ovaries, however, CWIN activity and *LIN5* expression did not increase until 2 DAP when fertilization occurred. Interestingly, a CWIN inhibitor gene *INVINH1* was repressed in the pollinated style at 2 DAP. In response to pollination, the style exhibited increased expressions of genes encoding hexose transporters, *SlHT1*, *2*, *SlSWEET5b*, and sucrose transporters *SlSUT1*, *2*, and *4* from 4 HAP to 2 DAP. Upon fertilization, *SlSUT1* and *SlHT1* and *2*, but not *SlSWEETs*, were also stimulated in fruitlets at 2 DAP. Together, the findings reveal that styles respond promptly and more broadly to pollination for activation of CWIN and sugar transporters to fuel pollen tube elongation, whereas the ovaries do not exhibit activation for some of these genes until fertilization occurs.

**Highlights:**

Expression of genes encoding cell wall invertases and sugar transporters was stimulated in pollinated style and fertilized ovaries in tomato.

## Introduction

In angiosperms, flowering signifies the onset of producing the next generation in the form of seeds embedded within fruit tissues. The stamen, the male organ of the flower, produces pollen grain that contains the male gamete-sperms. The pistil, the female organ of the flower, consists of stigma, style and ovary, which contains ovules carrying the female gamete-egg cells. At flowering stage, pollen grains from the stamen are transferred to the stigma aided by wind and animals (Regal, 1982) or insects such as bees, butterflies and moths (Regal, 1982; Free, 1993; Kremen et al., 2002; Lord and Russell, 2002), a process termed pollination. After landing on the stigma surface, pollen grains rehydrate to germinate as pollen tubes that carry the sperm cells to penetrate through the transmitting tissue of the style to the ovary. Upon reaching the ovules within the ovaries, the pollen tubes release the two sperm cells to complete the fertilization process (Heslop-Harrison, 1987; Mascarenhas, 1993; Franklin-Tong, 1999; Lord and Russell, 2002). The fertilized ovule and ovary then develop into seed and fruit, respectively. Thus, pollination and fertilization are the prerequisites for producing seeds. However, these processes are highly vulnerable to drought, heat and other abiotic stresses, which often leads to seed or fruit abortion, causing major crop yield loss (Boyer and Mclaughlin, 2007; Liu et al., 2016). Therefore, understanding the molecular players or pathways regulated by pollination and fertilization may help us identify valuable targets to improve reproductive success under stress.

After pollination, pollen grains germinate and elongate following a tip growth model at a rate up to 1 cm per hour, rendering it among the fastest growing cells (Lord and Russell, 2002). It takes hours to days for the pollen tube to reach the ovules for fertilization to occur (Taylor and Hepler, 1997). Structurally, the pollen tube is symplasmically isolated from the surrounding style tissue (Scott et al., 1991). Similarly, the filial tissues of angiosperm seeds, namely the embryo and endosperm, are isolated from the maternal tissue by an apoplasmic compartment (Stadler et al., 2005; Braun et al., 2014). Thus, all resources including sucrose, the predominant assimilate as carbon nutrient and energy source, must enter the elongating pollen tube and filial tissues apoplasmically (Reinders, 2016; Rottmann et al., 2016; Goetz et al., 2017).

In the apoplasmic unloading pathway, sucrose is unloaded into the cell wall matrix from the sieve element/companion cell (SE/CC) complex mediated by SWEET (sugars will eventually be exported transporter) proteins, which are typically localized on the plasma membrane to transport sucrose or hexoses down a concentration gradient in an energy independent manner (Chen et al., 2010; Eom et al., 2015). Within the cell wall, sucrose is often hydrolyzed by cell wall invertase (CWIN, with a pH optimum of 5–6) into glucose and fructose (Ruan et al., 2010). The hexoses are then taken up by H^+^-coupled hexose transporters (HXTs) (Ruan et al., 1995; Ruan et al., 2012; Ruan, 2014). It is common that CWIN and HXTs are co-expressed in the apoplasmic unloading region, indicating a synergistic functional relationship between CWIN and HXTs (Weber et al., 2005; Jin et al., 2009; McCurdy et al., 2010). It is less clear if the SWEETs for hexoses share a similar relationship with CWIN. Compelling evidence has demonstrated the importance of CWIN and sugar transporters in plant development. For instance, suppression of tomato hexose transporters, *SlHT1*, *2* or *3*, causes a 55% decrease in fruit hexose accumulation (McCurdy et al., 2010). A deficit in SWEET transporters led to defective seed and impaired pollen grains in *Arabidopsis* (Guan et al., 2008; Chen et al., 2015) and rice (Sosso et al., 2015; Yang et al., 2018). Similarly, decreased CWIN activity is associate with grain growth repression and abortion in maize (Mclaughlin and Boyer, 2004; Shen et al., 2018), rice (Hirose et al., 2002; Cho et al., 2005; Wang et al., 2008), and wheat (Dorion et al., 1996). Conversely, increasing CWIN activity by suppressing its inhibitor gene improved tomato fruit and seed set under normal and heat stress conditions (Jin et al., 2009; Liu et al., 2016). These findings demonstrated the important roles of CWIN and sugar transporters in fruit and seed development.

Despite the progress outlined above, it remains largely unknown as to how pollination and fertilization may alter the expression of CWIN and sugar transporters to power pollen tube elongation and seed and fruit set. Given assimilate is unloaded apoplasmically to the elongating pollen tubes and developing seeds, we hypothesize CWIN- and transporter-related sugar import and utilization may be enhanced during pollination and fertilization to support the transition from ovule to seed and ovary to fruit. To this end, the expression of CWIN gene *LIN5* was changed from a dispersed- to a phloem-specific pattern with CWIN activity dramatically increased in tomato ovaries from 2 days before anthesis to 2 days after anthesis (Palmer et al., 2015). These changes were proposed to be essential for the reproductive system to channel carbon nutrients more efficiently to the fertilized ovaries, named as a “Ready-Set-Grow” model (Palmer et al., 2015; Ru et al., 2017). It remains unknown, however, whether these changes were induced by the occurrences of pollination or fertilization. Historically, these two processes were suggested to affect several biological processes. For instance, the occurrence of pollination could induce petal and style withering (Van Doorn, 1997) or inhibit the elongation of maize silks as we observed in the field (Shen et al., 2018), while fertilization of the egg cell triggers endosperm proliferation in angiosperm embryogenesis (Nowack et al., 2006). Several recent studies have indicated the involvement of hexose transporters and CWIN in carbohydrate supply to the growing pollen tubes in *Arabidopsis* and tobacco (Reinders, 2016; Rottmann et al., 2016; Goetz et al., 2017). However, these studies did not differentiate the regulatory effect of pollination from fertilization on sugar transport and metabolism in the styles and fruitlets.

In this study, we aimed to dissect the potential effect of pollination and fertilization on the expression of genes encoding CWIN and sugar transporters in reproductive organs by using tomato (*Solanum lycopersicum*) as the experimental model. The choice of tomato was based on the following considerations: First, tomato is a major horticultural crop worldwide and a model system for studying fruit development. Thus, findings from tomato may be applicable to other fruit crops. Second, the tomato genome has been fully sequenced (Tomato Genome Consortium, 2012), with ample prior knowledge on genes controlling sugar transport, and metabolism (Godt and Roitsch, 1997; Fridman and Zamir, 2003; Proels et al., 2006). Third, it has been well established that, in tomato ovary, sucrose is unloaded apoplasmically (Jin et al., 2009; Palmer et al., 2015) where CWIN and sugar transporters play irreplaceable roles (Jin et al., 2009). We conducted emasculation and manual pollination treatments followed by sampling at specific time points to separately assess the effect of pollination and fertilization on the expression of genes for CWIN and sugar transporters.

## Materials and Methods

### Plant Growth Conditions

Tomato (*S. lycopersicum*) seeds were geminated on water-soaked filter paper at 25°C for 3 days. The seedlings were transferred to pots and grown in a greenhouse under a controlled day/night temperature regime of 25°C (6 am to 8 pm)/20°C with natural light illumination. Water and fertilizers were applied regularly as previously described (Liu et al., 2016; Ru et al., 2017).

### Emasculation, Manual Pollination, and Sampling

For emasculation treatment, the anthers of tagged flowers were removed at 2 days before anthesis (DBA). Care was taken to avoid damage to other parts of the flowers. The emasculated flowers were bagged to prevent natural pollination. On the day of anthesis (∼2 days after emasculation), manual pollination was conducted by brushing pollen grains from newly opened flowers to the styles of emasculated flowers. The manually pollinated flowers were then re-bagged. The styles and ovaries were manually dissected at 4 h and 2 days after pollination (HAP and DAP, respectively) and dropped into liquid nitrogen and storage at -80°C for further laboratorial measurements.

### Selection of Candidate CWIN, HXTs, SUTs, and SWEET Genes

There were 4 genes encoding CWIN in tomato (Godt and Roitsch, 1997). Among them, *LIN5* was the predominant member expressed in developing fruit and seed, and *LIN7* was restricted to the tapetum and pollen (Godt and Roitsch, 1997; Proels et al., 2006; Jin et al., 2009), whereas *LIN6* was expressed in all organs except the ovary and pollen, and *LIN8* was preferentially expressed in root (Fridman and Zamir, 2003). Since we focus on the style and ovary during pollination and fertilization processes, we selected *LIN5* and *7* as the candidate genes for CWIN based on their tissue-specific expression pattern.

There were 3 hexose transporter genes (*SlHT1*, *2* and *3*) identified from the tomato genome (Gear et al., 2000). *SlHT1* and *3* were dominantly expressed in green fruit, *SlHT2* was expressed at relatively high levels in source leaves and certain sink tissues (Gear et al., 2000; Dibley et al., 2005). Functionally, *SlHT1* and *2* are energy-dependent glucose transporters based on sugar uptake assay in yeast. *SlHT3* showed no sugar transport activity when expressed in yeast although its ortholog gene *AtSTP3* was demonstrated to be a low affinity transporter of glucose and probably other hexoses (Büttner et al., 2000; Reuscher et al., 2014). Based on the tissue-specific analysis of the three *SlHTs* in floral tissues (Supplementary Figure S1), we selected *SlHT1*, 2, and *3* to examine their response to pollination and fertilization in this study.

Members of sucrose transporter (SUT) families were well established as sucrose/H^+^ symporters (Kühn and Grof, 2010). The tomato SUT family consists of *SlSUT1*, *SlSUT2,* and *SlSUT4* (Reuscher et al., 2014); among them, *SlSUT1* and *4* were expressed ubiquitously and thought to be the main importers of apoplasmic sucrose into phloem (Weise et al., 2000; Sauer, 2007). *SlSUT2* was reported to be involved in pollen tube growth (Hackel et al., 2006; Reuscher et al., 2014). All three *SlSUTs* were investigated in this study.

A total of 29 putative *SlSWEET* transporter genes were identified from the tomato GDB database^1^ and the Solanaceae Genomics Network database^2^. Genes were assigned numbers *SlSWEET1-17* based on their positions on tomato chromosomes 1–12 (Feng et al., 2015). We further selected the *SlSWEET* genes that are highly expressed in flower buds and fully opened flowers based on the RNA-seq data, available in the Tomato Functional Genomic Database^3^ and analyzed by Feng et al. (2015). We finally selected 5 candidate *SlSWEET* genes that are preferentially expressed in flower organs, which were *SlSWEET5b*, *10a*, *11a*, *12a,* and *16*. Among them, *SlSWEET5b* belongs to clade II for transport of hexose, whereas *SlSWEET10a*, *11a,* and *12a* belong to clade III for transport of sucrose, and *SlSWEET16* belongs to clade IV transporters for hexose (Supplementary Figure S2). Phylogenetic analysis was conducted by comparing SWEET protein sequences in tomato and *Arabidopsis* by firstly conducting alignment of the SWEET amino acid sequences using ClustalW in Mega Alignment software followed by constructing an unrooted phylogenetic tree using Mega 7.0 with the neighbor-joining (NJ) method (Saitou and Nei, 1987). The settings of Poisson correction, pairwise deletion, and bootstrapping (1000 replicates; random seeds) served as default values. Based on the phylogenetic tree and prior knowledge from the literature, the locations and functions of the 5 candidate *SlSWEET* transporters were inferred.

### RNA Extraction and qRT-PCR

RNA was extracted using the RNeasy Plant Mini Kit (Qiagen). Genomic DNA was removed by using DNase (Promega). The DNase-treated RNA was reverse-transcripted into cDNA using oligo(dT)_20_ primer and ProtoScript Reverse Transcriptase (New England Biolabs) according to the manufacturer’s instructions. The cDNA was diluted 1:20 for quantitative real-time PCR (qRT-PCR) analyses according to Ru et al. (2017). Relative gene expressions of target genes were calculated by comparing with the reference genes of *SlTIP4* and *SlExpress* (Ru et al., 2017). Primers used for qRT-PCR are list in Supplementary Table S1.

### Invertase Enzyme Activities Assay

Cell wall bound and soluble proteins were extracted and assayed according to Tomlinson et al. (2004) and Palmer et al. (2015).

### Staining and Visualization of Pollen Tube Within the Pistils

Pollen tubes were visualized by staining with aniline blue, which gives a strong fluorescence under UV excitation after binding with callose in pollen and pollen tube (Martin, 1959). Following manual pollination on the day of anthesis, the pollinated pistils were sampled 24 and 48 HAP and fixed immediately in FAA solution (40% formaldehyde solution: 80% ethanol: acetic acid glacial = 1:8:1, v/v/v). The fixed pistils were then rinsed with distilled water for several times and transferred into 8 M NaOH solution for 24 h to soften the tissue (Martin, 1959). After neutralizing in acetic acid glacial for 30 s, the pistils were immersed in distilled water for 1 h to remove excess acetic acid. The pistils were stained with 0.1% aniline blue in 0.1 M K_2_HPO_4_-KOH (pH 11) for 4 h and then put on a glass slide. The pistils were evenly squashed by tapping the cover slip slightly with tweezers and then observed with a fluorescence microscope under UV.

### Statistically Analysis and Illustration Drawing

All measurements described comprised at least four biological replicates. Each biological replication contained 2 ovaries or 4 styles. Statistical analyses were conducted by using Student’s *t-test* in the EXCEL software (Microsoft). Illustrations were drawn by Microsoft PowerPoint 2016 and Adobe Photoshop CS6.

## Results

### Validation of Emasculation and Pollination Treatments

**FIGURE 1:**
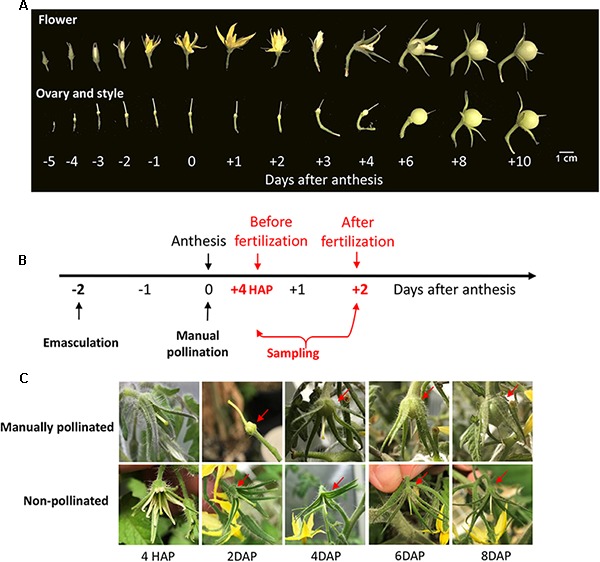
The phenotype of style and ovary during flowering stage in tomato and the illustration of time of manual pollination and sampling. **(A)** The phenotypes of tomato flower, ovary, and style from flower bud to fruitlets. The elongation of the style stopped at ∼2 days before anthesis. Fruitlet enlargement was not apparent until 3 DAP. **(B)** A schematic diagram about the timing of emasculation, pollination, and sampling. The ovary and style were harvested at 4 HAP and 2 DAP, representing the time points before and after fertilization, respectively. Emasculated flower with no pollination served as a control. **(C)** The phenotypes of manually pollinated ovary and style from 4 HAP to 8 DAP. The pollinated ovary increased its size at 2 DAP onward (red arrows). In contrast, the non-pollinated control ovaries were stunted and aborted at 6∼8 DAP. The findings validated the success of emasculation and pollination treatments. HAP, hours after pollination; DAP, days after pollination.

Under natural condition, the styles elongated from 5 days before anthesis (DBA) and reached its maximum at 2 DBA (Figure 1A), implying that the pistil was developmentally ready to receive pollen grains at 2 DBA. Following anthesis, the petals changed their color from yellow to pink and purple and then became welted, whereas the ovaries did not enlarge and the style did not show any sign of senescence until the 4th day after anthesis (DAA, Figure 1A).

To examine the impact of pollination on CWIN and sugar transporter gene expression, we performed emasculation at 2 DBA and manually pollinated the emasculated stigma on the day of anthesis, i.e., 0 DAA (Figure 1B). In tomato, it takes ∼48 h for the elongating pollen tubes to reach the ovules to complete fertilization (see below). Thus, we sampled styles and ovaries at 4 h after pollination (HAP) and 2 days after pollination (DAP), which represented the stages of before- and after-fertilization, respectively (Figure 1B). The manually pollinated ovaries showed visible enlargement from 4 DAP, the same as that under natural pollination, whereas non-pollinated ovaries did not (Figure 1A,C), indicating the successes of manual pollination, and emasculation, respectively.

### The Expression of CWIN Gene *LIN5* and CWIN Activity Were Enhanced in the Style and Ovary During Pollination and Fertilization, Respectively

**FIGURE 2:**
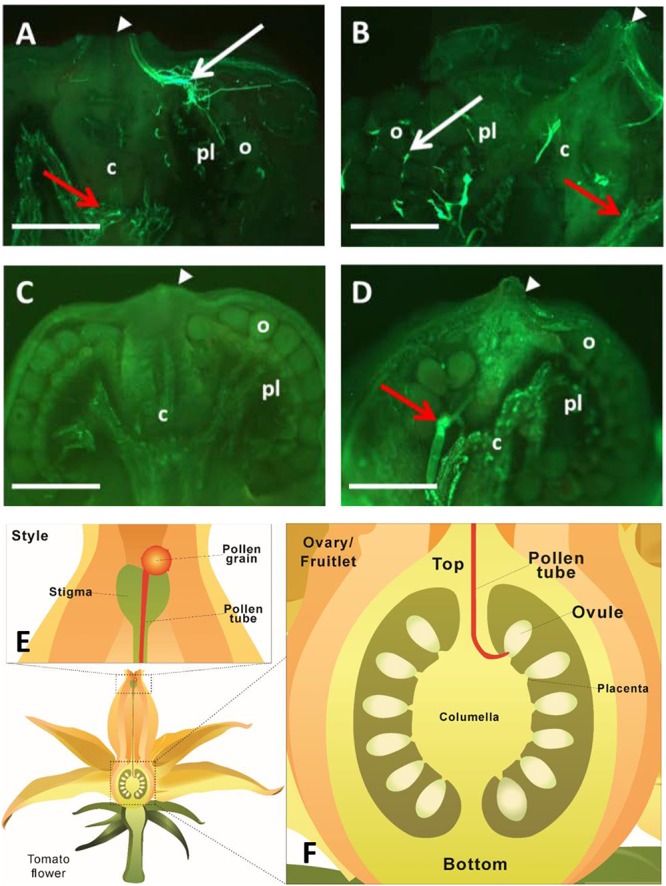
Visualization of pollen tube elongation within tomato pistils. **(A,B)** Longitudinal sections of ovary and fruitlet at 24 and 48 h after pollination (HAP), respectively. Pollen tubes, exhibiting fluorescence from the callose, entered the ovary through the junction of ovary and style (white arrowheads) at 24 HAP **(A)** and arrived at ovules at 48 HAP **(B)**, as indicated by white arrows. **(C)** Negative control I, fruitlet derived from pollinated flower at 48 HAP without staining with aniline blue. No fluorescence was detected in the fruit. **(D)** Negative control II, fruitlet at 48 HAP, derived from non-pollinated flower, but stained with aniline blue. Fluorescence was only found at the bottom of fruit, but not in ovules. Red arrows at the bottom of ovaries in **(A,B,D)** indicate fluorescence from the pre-existing callose of ovary tissues, mainly the vascular bundles. White arrowheads indicate the connection between ovary and style in **(A**–**D)**. Each image is one representative out of four biological replicates. c, columella; pl, placenta; o, ovule; Scale bars represent 0.25 mm in **(A,B)**, 0.5 mm in **(C,D)**. **(E)** An illustration showing that pollen tube elongates along the style. **(F)** A schematic diagram of a tomato ovary. The pollen tube enters through the top of the ovary and reaches ovules to finish the fertilization process.

To determine when fertilization likely occurred, we investigated pollen tube elongation *in vivo* within the style. The analyses revealed that pollen tubes emitting green-blue fluorescence from aniline blue staining of callose entered the ovary and reached ovules at 24 and 48 HAP (Figure 2A,B). Intriguingly, the fluorescence was not only found at the top of the ovary, but also at the bottom (Figure 2A,B). To check if the fluorescence was derived from pollen tubes, two negative controls were included. No fluorescence was found at either the top or bottom of the ovary in negative control I (Figure 2C), in which the ovary was derived from pollinated flower, but not stained with aniline blue, indicating the fluorescence in Figure 2A,B is callose-specific. In negative control II (Figure 2D), the ovary was derived from non-pollinated flower, but stained with aniline blue. Here, some fluorescence was observed at the bottom of the ovary, but with no fluorescence detected in the region where ovules were located (Figure 2D). These results indicate that the fluorescence at the top of the ovaries in Figure 2A,B comes from the callose of pollen tubes, whereas the fluorescence at the bottom of the ovaries originates from pre-existing callose of other ovary tissues, likely the vascular bundle in the placenta region. The key point here is that, compared with the negative controls with no fluorescence in the ovules (Figure 2C,D), pollination treatment showed fluorescence of pollen tubes reached the ovules by 48 HAP (Figure 2B) as illustrated in the schematic diagrams (Figure 2E,F). Thus, it can be concluded that most pollen tubes have entered into ovaries at 24 HAP and reached ovules at 48 HAP (Figure 2A,B), indicating that fertilization did not happen at 24 HAP but took place at 48 HAP under our experimental conditions.

**FIGURE 3:**
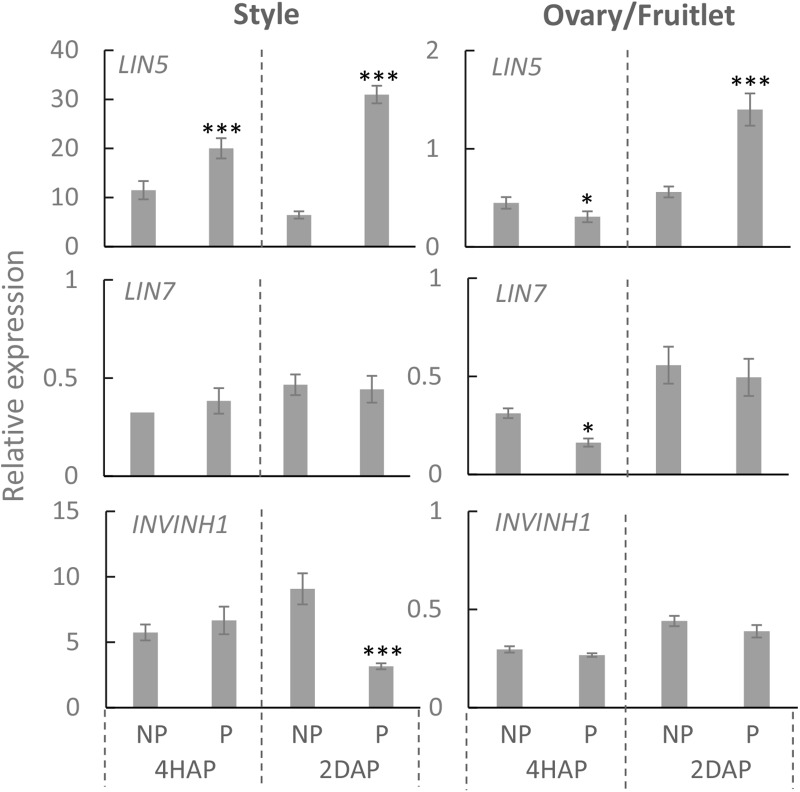
Relative expression of CWIN-related genes *LIN5*, *LIN7*, and *INVINH1*, in style and fruitlet in response to pollination at 4 HAP and fertilization at 2 DAP. Asterisks indicate significant differences (Student’s *t*-test, ^∗^*p* < 0.05; ^∗∗^*p* < 0.01; ^∗∗∗^*p* < 0.001, *n* = 4) between non-pollination and pollination treatments. DAP, days after pollination; HAP, hours after pollination.

Compared with non-pollinated treatment, the expression of *LIN5* was induced by ∼2 times at 4 HAP and ∼3 times at 2 DAP in the manually pollinated style (Figure 3). Interestingly, *LIN5* expression in the ovaries was not induced, but rather was slightly reduced by the pollination treatment at 4 HAP (Figure 3). Remarkably, the CWIN mRNA level was more than doubled in the ovaries by 2 DAP (Figure 3), when fertilization has occurred (Figure 2), turning the ovaries into fruitlets. By comparison, pollination treatment did not affect the expression of *LIN7*, which is mainly expressed in anthers and pollens (Godt and Roitsch, 1997), except a slight reduction in ovaries at 4HAP. The expression of *INVINH1* encoding the inhibitory protein against CWIN activity (Jin et al., 2009) was significantly reduced at 2 DAA in pollinated style compared with non-pollinated ones but not affected in the ovaries by pollination treatment at 4 HAP or 2 DAP (Figure 3).

**FIGURE 4:**
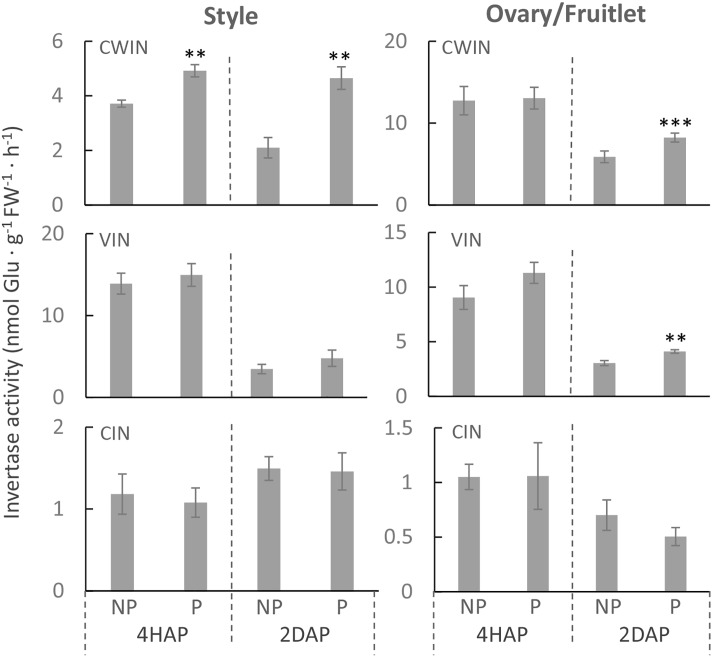
The activities of CWIN, VIN, and CIN in style and fruitlet in response to pollination at 4 HAP and fertilization at 2 DAP. Asterisks indicate significant differences (Student’s *t*-test, ^∗^*p* < 0.05; ^∗∗^*p* < 0.01; ^∗∗∗^*p* < 0.001, *n* = 4) between non-pollination and pollination treatments. DAP, days after pollination; HAP, hours after pollination.

Consistent with the gene expression data (Figure 3), an enzyme assay revealed that CWIN activity was significantly increased in the pollinated style from 4 HAP to 2 DAP and in the fertilized fruitlet at 2 DAP (Figure 4). In higher plants, there are other invertases hydrolyzing sucrose into hexoses, namely cytoplasmic invertase (CIN), and vacuolar invertase (VIN), classified based on their subcellular localizations (Ruan, 2014). Previous research has demonstrated that the VIN contributes to cell division and expansion, whereas the CIN may play a role in cytosolic hexose homeostasis (Ruan, 2014; Wan et al., 2018). In this experiment, the pollination-triggered increase in sucrose hydrolysis activity in the style appeared specific to CWIN since neither VIN nor CIN activity was induced during pollination and fertilization (Figure 4). Within the ovaries/fruitlets, VIN activity also showed a slight increase by the pollination treatment at 4 HAP and 2 DAP, with no changes in CIN activity (Figure 4).

### Expression of Hexose Transporter Genes, *SlHT1,2* and *SlSWEET5a*, Was Stimulated During Pollination in the Style With *SlHT1, 2* Expression Enhanced Upon Fertilization in the Fruitlets

**FIGURE 5:**
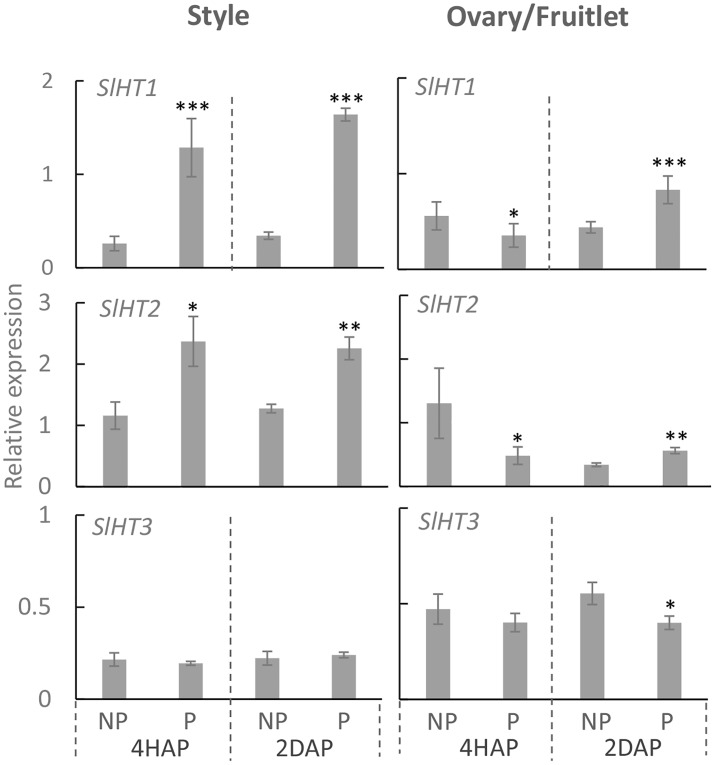
Relative gene expression of *SlHT1*, *SlHT2*, and *SlHT3* in style and fruitlet in response to pollination at 4 HAP and fertilization at 2 DAP. Asterisks indicate significant differences (Student’s *t*-test, ^∗^*p* < 0.05; ^∗∗^*p* < 0.01; ^∗∗∗^*p* < 0.001, *n* = 4) between non-pollination and pollination treatments. DAP, days after pollination; HAP, hours after pollination.

Three hexose transporter (HT) genes are expressed in developing tomato fruit, which are *SlHT1, 2,* and *3* (Gear et al., 2000). They belong to the STP sub-family of monosaccharide transporters that localize to the plasma membrane (McCurdy et al., 2010). Here, we found that in the pistil, only *LeHT1* and *3* were highly expressed from 2DBA to 4DAA (Supplementary Figure S1), whereas, all three *SlHT* genes were expressed in anther and petal (Supplementary Figure S1). Interestingly, the *SlHT1* transcript level was induced by ∼5 times in the pollinated style from 4 HAP to 2 DAP compared with the non-pollinated one (Figure 5). In the ovaries, the expression of *SlHT1* showed a slight reduction at 4 HAP prior to fertilization but a significant increase at 2 DAP upon fertilization (Figure 5). Although *SlHT2* was slightly expressed in pistil (Supplementary Figure S1), its expression was significantly increased in the pollinated style from 4 HAP to 2 DAP and in the fertilized ovary at 2DAP (Figure 5). *SlHT3* showed no response to pollination, except a slight reduction in the fertilized fruitlet at 2 DAP (Figure 5).

We then examined the possible effect of pollination and fertilization on the expression of a class of recently identified genes encoding SWEETs. Among the 29 *SWEETs* in the tomato genome, only *SlSWEET5b,* *10b*, *11a*, *12a,* and *16* were highly expressed in flower buds and fully opened flowers (Feng et al., 2015). Phylogenetic analysis of the tomato SWEETs with that in *Arabidopsis* (Supplementary Figure S2), coupled with current knowledge on the functionality of SWEETs (Chen et al., 2010; Chen et al., 2015; Eom et al., 2015), revealed that SWEET5b belonged to clade II for transport of hexoses, whereas SWEET10b, 11a, 12a were grouped with clade III for moving sucrose, and SWEET16 is a clade IV uniporter functioning on tonoplast for transport of hexoses.

**FIGURE 6:**
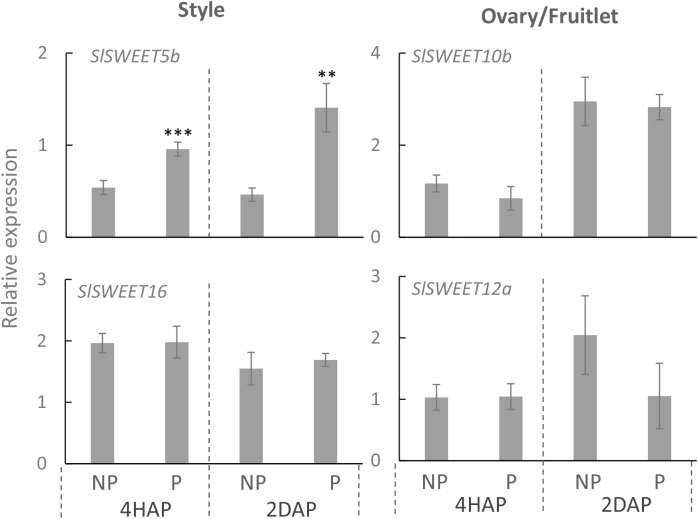
Relative expression of *SlSWEET* transporter genes in response to pollination at 4 HAP and fertilization at 2 DAP. Asterisks indicate significant differences (Student’s *t*-test, ^∗^*p* < 0.05; ^∗∗^*p* < 0.01; ^∗∗∗^*p* < 0.001, *n* = 4) between non-pollination and pollination treatments. DAP, days after pollination; HAP, hours after pollination.

Quantitative real-time PCR results showed that expressions of *SlSWEET5b* and *16* were increased in the style at 4 DAA compared with that at 2 DBA, whereas in the ovary or fruitlet *SlSWEET10b* and *12a* were highly expressed at 2 DBA and 4 DAA (Supplementary Figure S3). *SlSWEET11a* exhibited little expression in the style and ovary (Supplementary Figure S3), and hence is not included in our further analyses. Notably, in the style *SlSWEET5b* gene expression was significantly stimulated from 4 HAP to 2 DAP by the pollination treatment (Figure 6). This gene was not expressed in the ovary or style at 2 DBA (Supplementary Figure S3) and not responsive to pollination in the fruitlet. *SlSWEET10b* and *12a* were expressed in the ovaries and fruitlets (Supplementary Figure S3). However, neither of them responded to pollination or fertilization (Figure 6). *SlSWEET16* also did not show any difference between pollinated and non-pollinated treatments (Figure 6).

### Pollination Treatment Significantly Enhanced the Expression of *SlSUT1, 2*, and *4* in the Style but With Only S*lSUT1* Increased in the Ovaries Upon Fertilization

**FIGURE 7:**
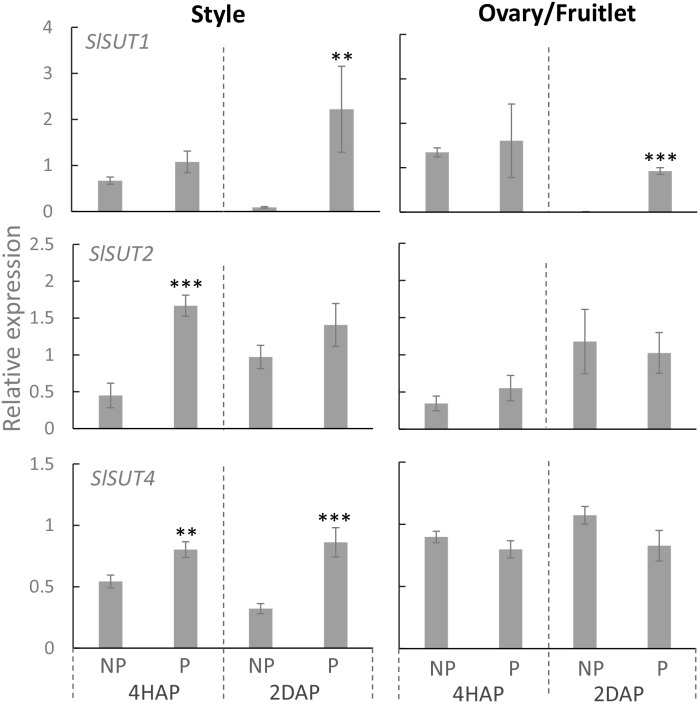
Relative expression of *SlSUT* transporter genes in response to pollination at 4 HAP and fertilization at 2 DAP. Asterisks indicate significant differences (Student’s *t-test*, ^∗^*p* < 0.05; ^∗∗^*p* < 0.01; ^∗∗∗^*p* < 0.001, *n* = 4) between non-pollination and pollination treatments. DAP, days after pollination; HAP, hours after pollination.

Proton-coupled sucrose transporters (SUTs) are also involved in the delivery of sugars to support pollen tuber elongation in tomato (Hackel et al., 2006), tobacco (Goetz et al., 2017), *Arabidopsis* (Rottmann et al., 2018), and rice (Hirose et al., 2014). We therefore examined if expression of *SlSUTs* in the style and ovary were impacted upon pollination and fertilization. In tomato, there are three *SIUT* genes expressed in floral bud and flowers, namely *SlSUT1*, *2*, and *4* (Hackel et al., 2006; Kühn and Grof, 2010). qRT-PCR analyses showed that *SlSUIT 2* and *4* were upregulated in the pollinated style at 4 HAP, followed by a dramatic increase in the mRNA levels of *SlSUT1* and *4* at 2 DAP (Figure 7). The ovaries did not respond to pollination at the transcript levels of the three *SlSUTs* at 4 HAP but exhibited an induction for *SlSUT1* at 2 DAP when fertilization occurred (Figure 7).

## Discussion

Pollination and fertilization are prerequisite for flower plants to set seed and fruit. These processes are highly energy-consuming and rely on the efficient input of sugars to fuel the growth of pollen tubes and the young seeds and fruitlets (Ruan et al., 2012). CWIN and sugar transporters are known to play crucial roles in supplying nutritional and signaling sugars into elongating pollen tubes, embryos, and endosperms that were symplasmically isolated from the surrounding maternal tissues (Wang and Ruan, 2012; Goetz et al., 2017). To our knowledge, however, there have been no reports which dissected the specific effects of pollination and fertilization on the expression of these genes. Here, we present evidence that the expression of CWIN and some specific sugar transporters was differentially stimulated in style and fruitlet upon pollination and fertilization, respectively. These findings provide insights into the roles of pollination and fertilization in activating apoplasmic sucrose unloading and input in reproductive organs and identify new genetic targets for improving reproductive success.

### CWIN Expression and Activity in the Style and Fruitlets Were Activated Upon Pollination and Fertilization, Respectively

It has been reported that by applying a chemical invertase inhibitor, miglitol, *in vitro* pollen germination and pollen tube growth were strongly inhibited, which could not be fully restored by adding sucrose into the medium (Goetz et al., 2017). This finding indicates the role of invertase in pollen germination and growth likely by facilitating apoplasmic unloading of sucrose to support heterotrophic pollen tube growth. Similarly, down-regulation of CWIN gene expression or activity correlates with pollen sterility and grain abortion in rice (Hirose et al., 2002; Oliver et al., 2005) and maize (Ruan et al., 2010; Shen et al., 2018). However, it remains largely unknown how CWIN and its functionally related genes respond to pollination and fertilization for seed and fruit set. To this end, the expression of *LIN5* was changed from a dispersed pattern in unfertilized ovaries to a phloem-specific pattern in young fruitlet, indicating pollination or fertilization could elicit a signal or signals to transcriptionally or post-transcriptionally modulate CWIN gene expression (Palmer et al., 2015). However, whether the signals were derived from pollination or fertilization remains unknown. Here, we found that in the style *LIN5* expression was stimulated by pollination treatment from 4 HAP to 2 DAP, whereas in the fertilized fruitlet *LIN5* expression was enhanced at 2 DAP when fertilization was accomplished (Figure 2, 3). These findings strongly indicate that the CWIN gene was stimulated by signals emitted during pollination and fertilization in the style and fruitlet, respectively, although the identity of the signals is yet to be determined. Moreover, we noticed that the CWIN inhibitor gene *INVINH1* was significantly suppressed in the style by pollination treatment at 2 DAP (Figure 3). Consequently, CWIN activity was significantly increased in the pollinated style and fertilized fruitlet (Figure 4). The elevated CWIN activity likely supports the rapid elongation of pollen tubes and the onset of seed and fruit development upon fertilization by (i) facilitating apoplasmic phloem unloading of sucrose in the style and in the fruitlets (Ruan et al., 1995; Palmer et al., 2015), and (ii) providing hexoses to fuel pollen tube elongation and to promote cell division in the newly formed fruitlets (Ruan et al., 2012).

### Activated *SlHT1* and *2* During Pollination and Fertilization Could Channel Hexoses to Pollen Tubes and Fertilized Fruitlets

After sucrose hydrolysis by CWIN, the resultant hexoses could be taken up into the cytoplasm of the recipient cells by H^+^-coupled HTs (Ruan and Patrick, 1995; Ruan, 2014). Among the three *SlHTs,* *SlHT1*, *2,* and *3* that were expressed in flowers and young fruits (McCurdy et al., 2010), *SlHT1* and *3* were highly expressed in ovary/fruitlet and the style whereas *SlHT2* was preferentially expressed in the anther and petal (Supplementary Figure S1), indicating their different roles in the flowering and fruit set processes.

*HTs* have been shown to be responsive to pathogens (Fotopoulos et al., 2003; Lemonnier et al., 2014), abiotic stresses (Yamada et al., 2011), and its substrate sugars (Rottmann et al., 2016; Rottmann et al., 2018). In this study, we found that *SlHT1* and *2*, but not *3*, were stimulated in the style after pollination and in the fruitlet upon fertilization (Figure 5). This finding complements the understandings on metabolic regulation on HT in reproductive organs. We hypothesize that the activated *SlHT1* during pollination and fertilization is significant for the growth of pollen tubes and fertilized fruitlets for the following reasons: (i) *SlHT1*, a member of the STP families, was localized on the plasma membrane to transport hexoses (McCurdy et al., 2010), and it would provide hexoses into the growing pollen tube and rapidly proliferating fruitlet as an energy source and signaling molecules (Ruan et al., 2010; Ruan, 2014; Goetz et al., 2017; Rottmann et al., 2018); (ii) the activated HT may help in maintaining the cellular osmotic potential and turgor that are required for cell elongation and enlargement (Ruan et al., 2001; Zhang et al., 2017); and (iii) HT may play regulatory roles by adjusting the cellular hexose concentration and thus sugar signaling pathways (Rolland et al., 2006; McCurdy et al., 2010; Ruan, 2014; Wan et al., 2018).

### *SlSUTs* in the Style Were Broadly Activated During Pollination but Less So in the Fruitlet Following Fertilization

In the processes of pollen germination and pollen tube elongation, SUT was demonstrated to operate in parallel with CWIN and HT (Lemoine et al., 1999; Goetz et al., 2017). In tomato, three *SlSUTs* have been identified, among them *SlSUT1* and *SlSUT2* were predominantly expressed in the pollen tubes as well as in the sieve elements of young fruitlets (Hackel et al., 2006), whereas *SlSUT4* was expressed mainly in the styles and ovaries (Weise et al., 2000; Hackel et al., 2006). Immunolocalization analyses demonstrated that all three SlSUT proteins are localized at the plasma membrane of sieve elements in tomato (Reinders et al., 2002; Krügel et al., 2008). Previous studies have shown the critical roles of SUTs in the development of pollen grains (Lemoine et al., 1999; Hirose et al., 2010), pollen tube elongation (Hackel et al., 2006; Hirose et al., 2014), and seed development (Patrick and Offler, 2001). However, it remains unknown from these studies how the *SUTs* in styles and ovaries are potentially regulated during pollination or fertilization. Here, we found that expressions of *SlSUT1*, *2* and *4* in the style were all stimulated by pollination treatment, but only *SlSUT1* was enhanced in the fruitlet when fertilization occurred (Figure 7). Combined with the tissue-specific gene expression and subcellular localization of SUTs as discussed above (Weise et al., 2000; Hackel et al., 2006), we propose that in the styles, *SlSUT4* is rapidly activated, followed by *SlSUT1 and 2* during pollination to uptake sucrose into the growing pollen tubes from the phloem unloading region in the style. In the fruitlet, on the other hand, *SlSUT1* is stimulated upon fertilization to uptake more apoplasmic sucrose to support the rapid cell proliferation of the young seed and fruitlet. The analysis concurs with previous findings (Hackel et al., 2006) that *SlSUT1* and *2* antisense plants exhibited smaller fruits and fewer seeds. Our findings demonstrated the significance of successful pollination and fertilization on stimulating SUTs to support the growth of pollen tubes and young seeds and fruits.

### *SlSWEET5b* Expression Was Stimulated in the Style During Pollination but With No *SlSWEETs* Activated in the Fruitlet Upon Fertilization

In recent years, SWEETs were characterized and localized on the plasma membranes or tonoplasts to transport sucrose or hexoses, thereby regulating carbon allocation throughout the plant body (Chen et al., 2012; Eom et al., 2015). For instance, phloem loading was blocked and starch was accumulated in the *Atsweet11;12* double mutant (Chen et al., 2012), whereas phloem unloading and post-phloem transport were impaired in the *Arabidopsis sweet11;12;15* triple mutant, maize *Zmsweet4c* mutants and rice *Ossweet4* mutant, all leading to defective seed filling (Chen et al., 2015; Sosso et al., 2015; Ma et al., 2017).

Findings from our study provide new information regarding the impacts of pollination and fertilization on *SWEET* expression in reproductive organs. We found that in the tomato ovary and fruitlet *SlSWEET10b* and *12a* were the main ones expressed, whereas in style *SlSWEET5b* and *16* were dominant (Supplementary Figure S3). While *SlSWEET10b* and *12a* did not respond to fertilization in the fruitlets, the expression of *SlSWEET5b* in the style was specifically stimulated during pollination at 4 HAP and was further enhanced at 2 DAP (Figure 6). Phylogenetic analysis revealed SlSWEET5b belongs to clade II of the SWEET subfamily that was predicted to localize on the plasma membrane to transport hexoses (Supplementary Figure S2). *SlSWEET5b* was not expressed in the styles at 2 DBA when there were no pollen grains or pollen tubes (Supplementary Figure S3). Thus, it is apparently induced by pollination and likely functions in the uptake of apoplasmic hexoses into pollen tubes or reversely exported intracellular hexoses out of the pollen tubes (Chen et al., 2012). This bidirectional transport of hexoses is probably required for keeping cytosolic sugar homeostasis (Wan et al., 2018). We hypothesize that the coexistence of SlHTs (energy-dependent) and SlSWEET5b (energy-independent) may be crucial for maintaining fundamental cellular metabolism and function (Ruan, 2014). To this end, co-expression of energy-dependent and -independent sucrose transporters was observed in rapidly elongating cotton fibers, single-celled seed trichomes, by Zhang et al. (2017). Furthermore, a recent study has shown that an excess of cytosolic glucose inhibited pollen tube elongation (Rottmann et al., 2018), which further supports the concept that a system for bidirectional transport of sugars into and out of cytoplasm is crucial for cytosolic sugar homeostasis and cell viability (Ruan, 2014; Wan et al., 2018). Our finding, together with that reported by Rottmann et al. (2018), thus strongly indicates that the operation of an efflux pathway for excessive glucose is necessary for pollen tube growth.

### A Model on Rregulation of CWIN and Sugar Transporters During Pollination and Fertilization for Fruit and Seed Set

**FIGURE 8:**
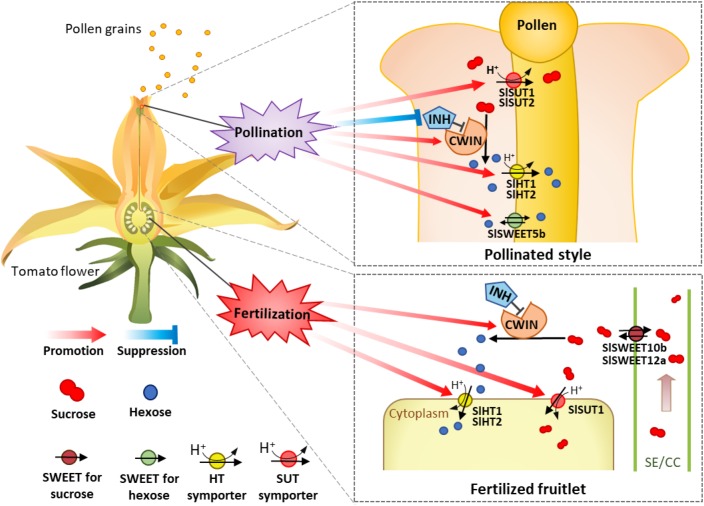
A model of how sugars transported into and metabolism within tomato style and ovary are regulated during pollination and fertilization. The occurrence of pollination stimulates and repressed cell wall invertase and its inhibitor gene (*INVINH1*), respectively, in the style, leading to high CWIN activity to hydrolyze sucrose into hexoses in the style apoplasm. Meanwhile, the expressions of sucrose transporter genes *SlSUT1* and *2* as well as *SlHT1* and *2* and *SWEET5b* for hexoses are also stimulated. The increased SlSWEET5b on the pollen tube might function in the uptake of apoplasmic hexose into pollen tubes or inversely exported intercellular hexose out of the pollen tube. The coexistences of SlSWEET and SlHT may contribute to the maintenance of cytosolic sugar homeostasis, which is crucial for metabolism and cellular function. These pollination-elicited changes enhance carbon input into and uses within pollen tubes to fuel their rapid elongation. For the ovaries or fruitlets, instead of pollination effect, the accomplishment of fertilization selectively stimulates the expression of genes for CWIN (*LIN5*), SlHT (*SlHT1* and *2*), and SlSUT (*SlSUT1*), but not those for SlSWEETs (*SlSWEET10b* and *12a*), to support the growth of the newly formed fruitlets.

Based on the analysis above, we propose a model of how the expression of CWIN and sugar transporters are regulated during pollination and fertilization to enhance assimilate input and utilization to fuel pollen tube elongation and seed and fruit set (Figure 8). In this model, CWIN and sugar transporters, differentially regulated during pollination and fertilization in the styles and fruitlets, are spatiotemporally coordinating with each other and collectively contribute to the uptake of apoplasmic sugars into pollen tubes and fertilized ovules to fuel their growth, characterized by rapid cell elongation in the former and cell division in the latter. Experiments are underway to identify the exact signals emitted from elongating pollen tubes or that are released from sperms or newly formed embryo and endosperm that activate the expression of CWIN and sugar transporter genes in the pollinated styles and fertilized ovules, respectively.

## Author Contributions

Y-LR conceived the project. SS and Y-LR designed the experiments. SS, SM, YL, SL, JL, LW, H-PM, and DK conducted the experiments. SS, SM, and Y-LR analyzed the data and wrote the manuscript with inputs from LW and H-PM.

## Conflict of Interest Statement

The authors declare that the research was conducted in the absence of any commercial or financial relationships that could be construed as a potential conflict of interest.

## Footnotes

1 http://www.plantgdb.org/

2 https://solgenomics.net/

3 http://ted.bti.cornell.edu/cgi-bin/TFGD/digital/home.cgi

## Supplementary Material

FIGURE S1 Relative expression of hexose transporter genes, *SlHT1*, *SlHT2,* and *SlHT3*, in different flower tissues of tomato at 2 days before (2 DBA) and 4 days after anthesis (4 DAA). Asterisks indicate significant differences (Student’s *t*-test, ^∗^*p* < 0.05; ^∗∗^*p* < 0.01; ^∗∗∗^*p* < 0.001, *n* = 4) between 2 DBA and 4 DAA.

FIGURE S2 Phylogenetic analysis of SlSWEET families in tomato in relation to *Arabidopsis*. Based on the current knowledge, SWEETs in clade I and clade II transport hexoses; while those in clade III transport sucrose, all are predicted to be located on plasma membrane. SWEETs in clade IV are located on tonoplast to transport hexose.

FIGURE S3 Relative expression of *SlSWEET* transporter genes, *SlSWEET5b*, *10b*, *11a*, *12a,* and *16*. Asterisks indicate significant differences (Student’s *t*-test, ^∗^*p* < 0.05; ^∗∗^*p* < 0.01; ^∗∗∗^*p* < 0.001, *n* = 4) between 2 DBA and 4 DAA.

TABLE S1 The primers used in this study.
