# Supplementary material for: Cell Wall Invertase and Sugar Transporters Are Differentially Activated in Tomato Styles and Ovaries During Pollination and Fertilization
Source: Front Plant Sci. 2019 Apr 18;10:506. doi: 10.3389/fpls.2019.00506 (PMC6482350; doi:10.3389/fpls.2019.00506)
Supplement: FIGURE S1 — Relative expression of hexose transporter genes, SlHT1, SlHT2, and SlHT3, in different flower tissues of tomato at 2 days before (2 DBA) and 4 days after anthesis (4 DAA). Asterisks indicate significant differences (Student’s t-test, ∗p < 0.05; ∗∗p < 0.01; ∗∗∗p < 0.001, n = 4) between 2 DBA and 4 DAA. [file Presentation_1.PPTX]

## Slide 1
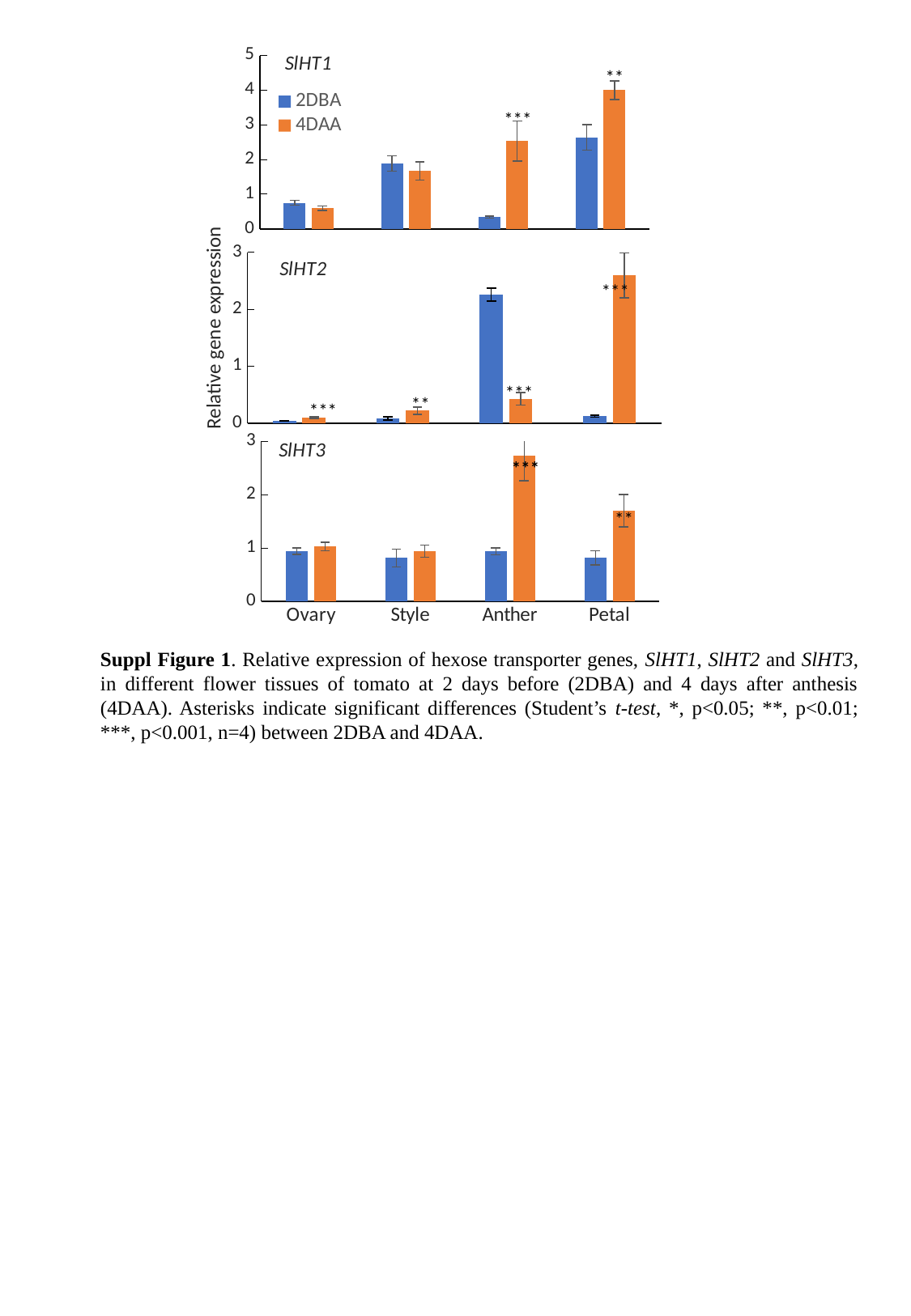

### Chart: SlHT1
| Category | 2DBA | 4DAA |
|---|---|---|
| Ovary | 0.7525409711607555 | 0.5991558368961926 |
| Style | 1.8850018760361107 | 1.664060513190135 |
| Anther | 0.3396339259652578 | 2.532494689471343 |
| Petal | 2.6339597049017547 | 3.9977362031032793 |**
***
### Chart: SlHT2
| Category | 2DBA | 4DAA |
|---|---|---|
| Ovary | 0.042434213236295364 | 0.09483107335811017 |
| Style | 0.07898452145818592 | 0.21681990736245807 |
| Anther | 2.256957575972916 | 0.4255750456300989 |
| Petal | 0.12155391498231102 | 2.594896264681786 |***
Relative gene expression
***
**
***
### Chart: SlHT3
| Category | 2DBA | 4DAA |
|---|---|---|
| Ovary | 0.9408592670727152 | 1.027213295024639 |
| Style | 0.8142984613256113 | 0.9414252364677987 |
| Anther | 0.9374691404379139 | 2.728745746168928 |
| Petal | 0.8163666959776753 | 1.6989297528238945 |***
**
Suppl Figure 1. Relative expression of hexose transporter genes, SlHT1, SlHT2 and SlHT3, in different flower tissues of tomato at 2 days before (2DBA) and 4 days after anthesis (4DAA). Asterisks indicate significant differences (Student’s t-test, *, p<0.05; **, p<0.01; ***, p<0.001, n=4) between 2DBA and 4DAA.

## Slide 2
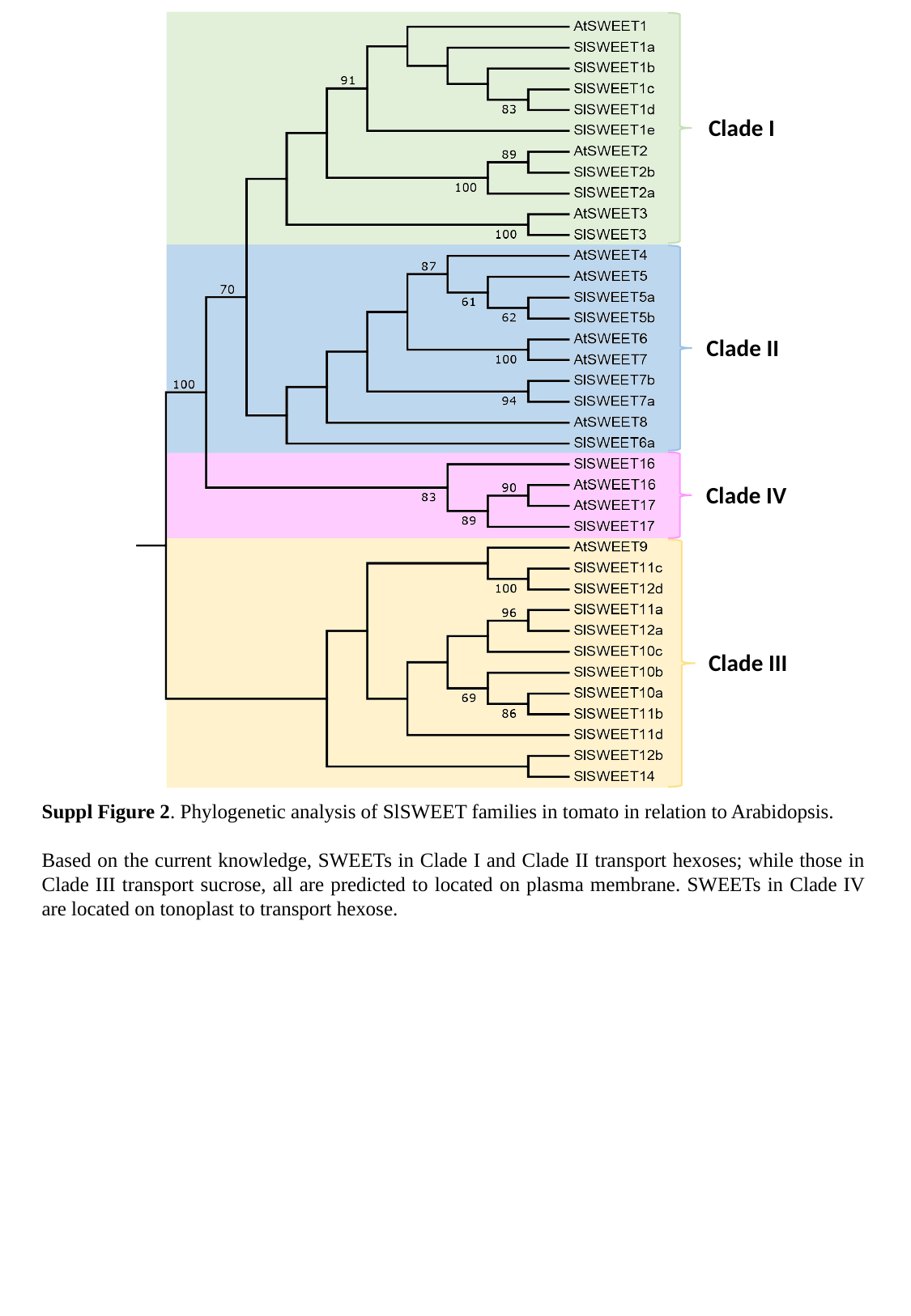

Clade I
Clade II
Clade IV
Clade III
Suppl Figure 2. Phylogenetic analysis of SlSWEET families in tomato in relation to Arabidopsis.
Based on the current knowledge, SWEETs in Clade I and Clade II transport hexoses; while those in Clade III transport sucrose, all are predicted to located on plasma membrane. SWEETs in Clade IV are located on tonoplast to transport hexose.

## Slide 3
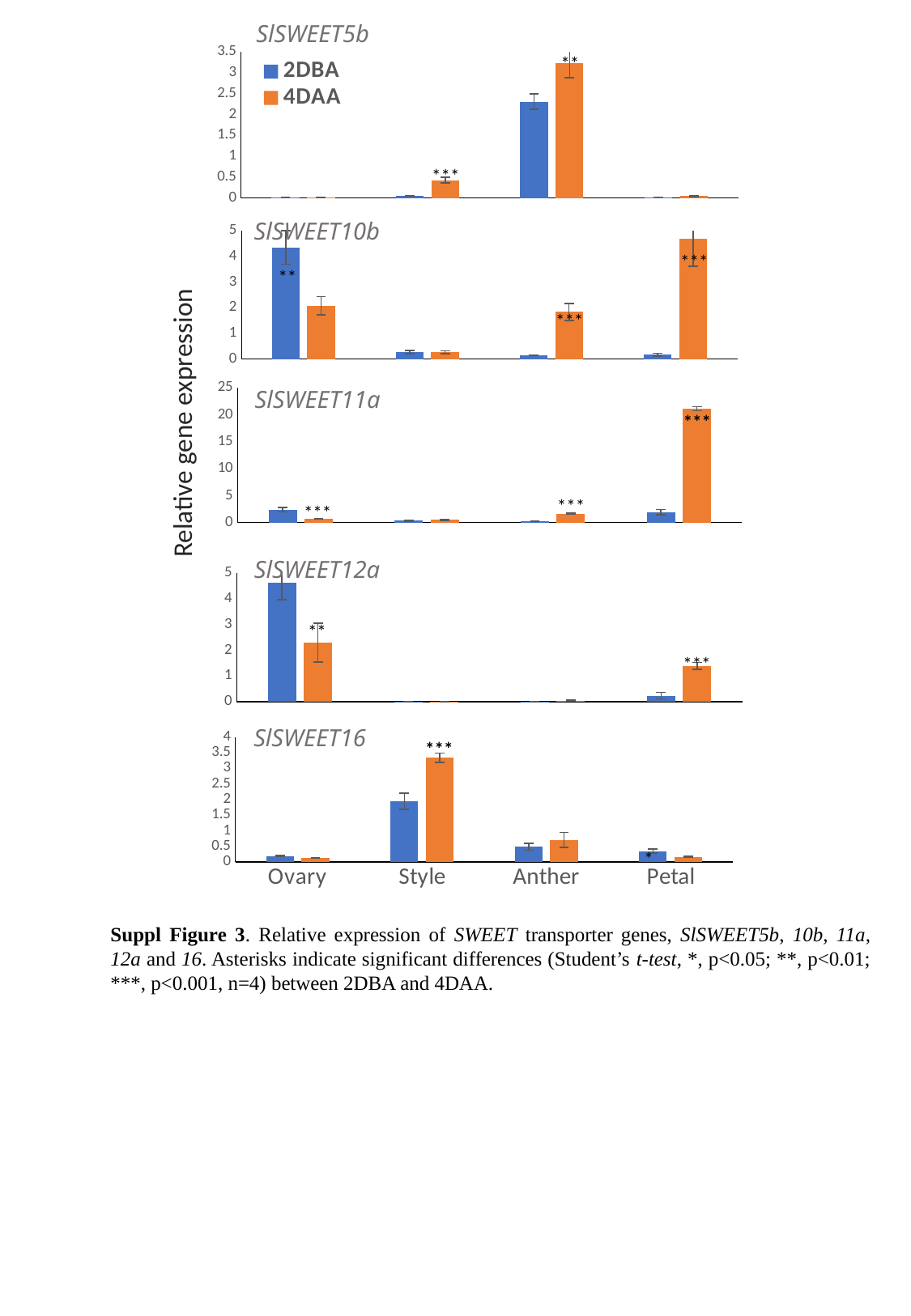

SlSWEET5b
### Chart
| Category | 2DBA | 4DAA |
|---|---|---|
| Ovary | 0.01031763947318478 | 0.007268890308825582 |
| Style | 0.0472673051758823 | 0.4260022001656058 |
| Anther | 2.3048183442763532 | 3.227314892937585 |
| Petal | 0.01839315972468282 | 0.040600994510337815 |**
***
SlSWEET10b
### Chart
| Category | 2DBA | 4DAA |
|---|---|---|
| Ovary | 4.3436857083353235 | 2.0708700466289964 |
| Style | 0.27503004917894125 | 0.2633096902566905 |
| Anther | 0.1517899235826205 | 1.8312575196113776 |
| Petal | 0.16867385774347415 | 4.679148709750882 |***
**
***
SlSWEET11a
### Chart
| Category | 2DBA | 4DAA |
|---|---|---|
| Ovary | 2.3903333606660553 | 0.7120879478151361 |
| Style | 0.31604947946964085 | 0.4812126818722277 |
| Anther | 0.2219165703097561 | 1.5924514666990623 |
| Petal | 1.9055439323676873 | 21.122396177759022 |***
***
***
Relative gene expression
SlSWEET12a
### Chart
| Category | 2DBA | 4DAA |
|---|---|---|
| Ovary | 4.621766166982186 | 2.2988002097556404 |
| Style | 0.003611151157094673 | 0.002847261332136289 |
| Anther | 0.003963252301800815 | 0.04038598881304278 |
| Petal | 0.23256173646998263 | 1.3926659247185091 |**
***
SlSWEET16
### Chart
| Category | 2DBA | 4DAA |
|---|---|---|
| Ovary | 0.1856954255008951 | 0.12699599025266273 |
| Style | 1.9398367991962968 | 3.3400144967947494 |
| Anther | 0.48140324821910385 | 0.6984198460944322 |
| Petal | 0.33927417444187447 | 0.1636352250738039 |***
*
Suppl Figure 3. Relative expression of SWEET transporter genes, SlSWEET5b, 10b, 11a, 12a and 16. Asterisks indicate significant differences (Student’s t-test, *, p<0.05; **, p<0.01; ***, p<0.001, n=4) between 2DBA and 4DAA.
